# Can Doppler ultrasound reduce hemorrhagic complications in ultrasound-guided percutaneous nephrolithotomy for solitary kidney stones?

**DOI:** 10.3389/fsurg.2025.1671428

**Published:** 2025-09-09

**Authors:** Bo Xiao, Xue Zeng, Yang Chen, Shen Lin, Yangyang Xu, Haiwen Huang, Zhichao Luo, Wenjie Bai, Jianxing Li

**Affiliations:** Department of Urology, Tsinghua University Affiliated Beijing Tsinghua Changgung Hospital, Beijing, China

**Keywords:** Doppler, percutaneous nephrolithotomy, solitary, kidney, bleeding

## Abstract

**Purpose:**

To evaluate the role of Doppler ultrasound in reducing hemorrhagic events during percutaneous nephrolithotomy (PCNL) in solitary kidney calculi through a comparative analysis.

**Patients and methods:**

We retrospectively reviewed the charts of urolithiasis patients who had undergone ultrasound-guided PCNL in our department from March 2021 to December 2024 and identified 76 eligible patients. Patients were stratified into two cohorts based on Doppler flow detection technology application: Group 1 (Doppler-mode, *n* = 29) and Group 2 (conventional mode, *n* = 47). Relevant patient characteristics, operative variables, and postoperative data were collected and analyzed, focusing on bleeding complications and stone-free rate (SFR).

**Results:**

No significant difference was found between the two groups regards to basic characteristics (gender, age, body mass index, stone size, pre-operative serum creatinine). The puncture time shows significant longer in Group 1 compared to Group 2 (173.1 ± 39.6 vs. 111.4 ± 29.9 s, *p* = 0.02). However, the hemoglobin loss reveals no significant difference between the two groups (17.5 ± 5.6 vs. 21.7 ± 6.3 g/L, *p* = 0.19). There were no significant differences in the operation duration (*p* = 0.47), transfusion rate (*p* = 0.15), postoperative creatinine (*p* = 0.80), overall complication (*p* = 0.07), final stone-free rates (*p* = 0.38) between the two groups.

**Conclusion:**

Doppler flow technology fails to confer superior benefits in ultrasound-guided PCNL for solitary renal calculus, with no definitive necessity established for hemorrhage risk mitigation.

## Introduction

Percutaneous nephrolithotomy (PCNL), established as the cornerstone for managing large (>2 cm) or staghorn renal calculi, achieves stone-free rates exceeding 90% in experienced hands (1). However, its invasiveness predisposes patients to risks such as bleeding (5%–23%) and visceral injury, particularly in anatomically challenging scenarios (2). These risks are magnified in solitary kidney patients (3). Physiological changes in solitary kidney include increased effective renal plasma flow, glomerular ultrafiltration coefficient, leading to increased single-nephron GFR, glomerular hyperfiltration. Aggravated glomerular hyperfiltration may resulting in afferent arteriole dilation and leading to intraglomerular hypertension (4, 5). Surgeons' primary concerns regarding PCNL in patients with solitary kidneys revolve around the heightened risk of intraoperative hemorrhage and the potential for postoperative deterioration of renal function.

Previous studies have reported the application of PCNL in patients with solitary kidney stones under fluroscopy (6, 7). Ultrasound (US), as a safe and convenient imaging modality, has gained increasing acceptance among urologists for renal access guidance. The Doppler flow mode integrated into ultrasound systems allows real-time visualization of renal blood flow distribution, enabling surgeons to detect vascular-rich regions within the renal parenchyma at potential puncture sites (8). Prior research has demonstrated that leveraging Doppler functionality during PCNL reduces perioperative blood loss and the incidence of severe hemorrhage (9). However, there remains a paucity of evidence regarding its utility specifically in solitary kidney cases. This study aims to evaluate the efficacy and safety of Doppler-assisted ultrasound guidance in PCNL for patients with solitary kidneys, based on a retrospective analysis of a large contemporary cohort from our institution.

## Patients and methods

### Study design and population

Of the 76 patients with solitary kidney stones underwent US-guided PCNL from March 2021 to December 2024. A solitary kidney refers to either: anatomic absence (congenital or acquired unilateral renal agenesis), or functional solitary status characterized by profound contralateral renal function loss (GFR < 10% of total baseline renal capacity). Exclusion criteria: patients with a history of PCNL or open renal surgery. Patients were stratified into two cohorts based on the intraoperative application of Doppler flow mode for puncture guidance: the Doppler-assisted group (*n* = 29) and the conventional ultrasound group (*n* = 47). Ethical approval was granted by the Institutional Review Board (IRB) of Beijing Tsinghua Changgung Hospital.

### Surgical technique

**Figure 1:**
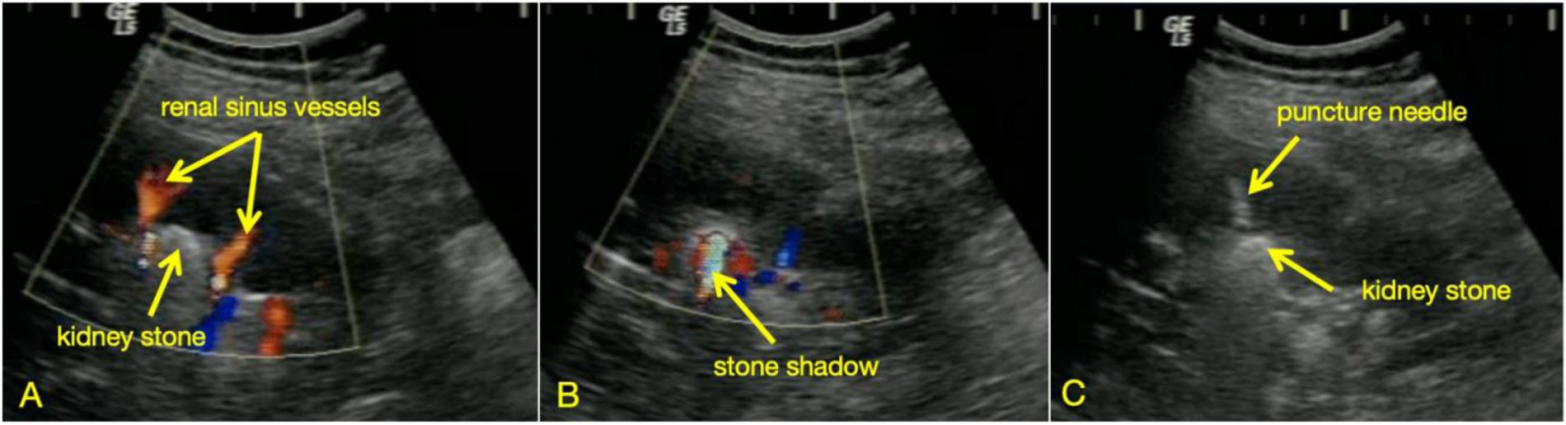
**(A)** Dopplar-mode shows preserved perfusion in the renal columns (normal vasculature), but hypovascularity at the fornix. **(B)** A prominent posterior acoustic shadow is noted deep to the calculus, suggesting possible adjacent prominent vasculature (originating from the renal columns) near the stone. **(C)** Switch off Doppler to optimize needle visibility during puncture.

All procedures were performed under general anesthesia. The patient was initially placed in the lithotomy position, and a 5Fr ureteral catheter was inserted retrograde into the affected renal pelvis. The distal end of the catheter was connected to a saline bag suspended at 100 cm height, secured to the urinary catheter for continuous infusion to dilate the renal pelvis and optimize puncture visualization. The patient was then repositioned to the prone position (or lateral decubitus position if prone positioning was intolerable). A 3.5 MHz convex ultrasound probe was used to scan the kidney. For the Doppler ultrasound group: the target calyx was first selected based on the operator's initial judgment. Doppler flow mode was activated to identify the site with minimal blood flow for puncture, eliminating reliance on subjective operator experience for optimal puncture positioning (Figure 1). For the conventional ultrasound group: puncture site selection relied entirely on the operator's subjective interpretation of B-mode ultrasound imaging and clinical experience. Both groups utilized the “three 30-degree triangulation method” described in our prior study (10) to select the target calyx, with needle insertion from the cephalad aspect of the ultrasound probe in the same plane. A 17.5G puncture needle (Urotech, Germany) was used. Upon successful puncture, a J-tipped guidewire was advanced into the target calyx under real-time ultrasound guidance. A 24Fr or 22Fr percutaneous nephrostomy tract was then established using an Alken coaxial metal dilator (Richard Wolf, Germany) or a high-pressure balloon dilator (X Force® N30, Bard, USA). The LithoClast® ultrasound/pneumatic lithotripsy system (Switzerland) or a high-power holmium laser was employed for stone fragmentation and clearance. The surgeon decided whether to terminate the procedure or create additional tracts based on intraoperative findings. Upon completion, a 14Fr or 20Fr nephrostomy tube was placed in each tract, and a 6Fr double-J stent was inserted for 2–4 weeks. The nephrostomy tube was removed 3–5 days postoperatively.

Stone size was defined as the maximum diameter of the dominant stone. For multiple stones, the sum of their maximum diameters was calculated. Stone-free status was assessed via non-contrast CT 1 month postoperatively and classified as: Grade A (no residual stones): absolute stone-free. Grade B (Grade A + fragments ≤2 mm): relative stone-free. Grade C (residual fragments 2.1–4 mm): fragmentary stone-free status. Estimated blood loss was determined by hemoglobin decline at 24 h postoperatively. Perioperative complications were graded using the modified Clavien-Dindo classification system.

### Statistical analysis

Continuous data are shown as the mean (standard deviation) and were compared between the study groups by one-way analysis of variance. Categorical data are shown as the number and/or percentage and were analyzed using the *χ*^2^ test or Fisher's exact test. All statistical analyses were performed using SPSS version 20.0 (IBM Corp., Armonk, NY, USA). A *p* value <0.05 was considered statistically significant.

## Results

The study cohort comprised 76 patients (29 in the Doppler-assisted group and 47 in the conventional ultrasound group) undergoing ultrasound-guided PCNL for solitary kidney stones. Baseline patient characteristics, including age, sex ratio, BMI, stone size, stone classification, preoperative serum creatinine, and prior renal surgery history, demonstrated no statistically significant differences between the two groups (*p* > 0.05, Table 1). The Doppler-assisted group exhibited significantly longer mean puncture time compared to the conventional group (173.1 ± 39.6 vs. 111.4 ± 29.9 s, *p* = 0.02). However, no significant differences were observed in operative duration (89.6 ± 20.7 vs. 97.7 ± 19.1 min, *p* = 0.47), hemoglobin decline (17.5 ± 5.6 vs. 21.7 ± 6.3 g/L, *p* = 0.19), or transfusion rates (3.4% vs. 4.3%, *p* = 0.15). Final stone-free rates (SFRs) showed comparable outcomes between groups (Grade A/B/C classification: 75.9% vs. 80.9%, *p* = 0.38). Postoperative serum creatinine levels (2.3 ± 0.5 vs. 2.2 ± 0.6 mg/dl, *p* = 0.80) and hospitalization duration (6.5 ± 3.3 vs. 7.1 ± 3.2 days, *p* = 0.53) were also similar. Both groups demonstrated comparable safety profiles, with low rates of major complications (Clavien > 2: 6.9% vs. 4.3%, *p* = 0.07). Minor complications (Clavien ≤ 2) occurred in 65.6% of Doppler cases and 53.2% of conventional cases (*p* = 0.07), predominantly transient hematuria and postoperative fever.

**Table 1:** Cohort patient characteristics.

Variables	Doppler-mode (*n* = 29)	Conventional mode (*n* = 47)	*p* value
Age (years), mean (SD)	49.5 (11.9)	46.8 (13.1)	0.69
Sex ratio (F/M)	14/15	21/26	0.24
BMI (kg/m^2^), mean (SD)	26.6 (5.8)	27.1 (7.2)	0.71
Stone size (cm), mean (SD)	5.3 (1.4)	5.9 (1.7)	0.51
Classification, *n* (%)			0.37
Congenital	15 (51.7)	26 (55.3)	
Acquired	14 (48.3)	21 (44.7)	
Urine culture, *n* (%)			0.17
Positive	18 (62.1)	31 (66.0)	
Negative	11 (37.9)	16 (34.0)	
Target calyx, *n* (%)			0.12
Upper	8 (27.6)	14 (29.8)	
Middle	17 (58.6)	29 (61.7)	
Lower	4 (13.8)	4 (8.5)	
Past renal surgery, *n* (%)			0.30
Yes	16 (55.2)	24 (51.1)	
No	13 (44.8)	23 (48.9)	
Puncture time (s), mean (SD)	173.1 (39.6)	111.4 (29.9)	0.02
Operative time (min), mean (SD)	89.6 (20.7)	97.7 (19.1)	0.47
Number of tracts, *n* (%)			0.49
Single	14	21	
Multiple	15	26	
SFR, *n* (%)			0.38
Grade A	12 (41.3)	19 (40.4)	
Grade B	16 (55.2)	29 (61.7)	
Grade C	22 (75.9)	38 (80.9)	
Mean decline in Hb level, g/L, mean (SD)	17.5 (5.6)	21.7 (6.3)	0.19
Blood transfusion rate, *n* (%)	2 (6.9)	3 (6.4)	0.15
Baseline serum creatinine, mg/dl, mean (SD)	2.4 (0.6)	2.1 (0.7)	0.21
Post-operative serum creatinine, mg/dl, mean (SD)	2.3 (0.5)	2.2 (0.6)	0.80
Post-operative hospitalization, days, mean (SD)	6.5 (3.3)	7.1 (3.2)	0.53
Complications, *n* (%)			0.07
Clavien ≤ 2	19 (65.6)	25 (53.2)	
Clavien > 2	2 (6.9)	2 (4.3)	
Stone composition, *n* (%)			0.53
Calcium oxalate	19	29	
Non-calcium oxalate	10	17	

## Discussion

US-guided PCNL has gradually gained popularity these years, offering core advantages such as zero radiation exposure, real-time dynamic imaging, and multiplanar visualization. Compared to traditional fluoroscopic guidance, US not only eliminates cumulative ionizing radiation risks for patients and operators but also provides clear visualization of peri-renal organs (e.g., bowel, liver), significantly reducing visceral perforation rates (11). However, the application of US in PCNL is constrained by two major challenges: First, steep learning curve, operators must master the spatial relationship between renal parenchyma and the collecting system, particularly in complex anatomic scenarios (e.g., narrowed renal pelvis, staghorn calculi) (12). Second, limited vascular discrimination, conventional B-mode US lacks resolution for detecting microvascular structures (e.g., interlobar arteries), relying on operator experience to avoid vascular-dense regions. The introduction of Doppler flow technology theoretically addresses this limitation. Doppler ultrasound visualizes blood flow information in real time by detecting frequency shifts caused by erythrocyte movement, displayed as color-coded maps (Color Doppler) or spectral waveforms (Spectral Doppler). Its primary applications include evaluation of renal artery stenosis, monitoring transplant renal function and guides avoidance of vascular-dense regions during renal biopsies. Doppler also enables real-time tracking of arteriovenous distributions around needle trajectories, especially valuable for anatomical variants (13, 14).

The management of stones in solitary kidney remains a challenging scenario, shock wave lithotripsy (ESWL) and flexible ureteroscopy (f-URL) have a acceptable stone free rate and simple procedure in small stones (15). However, PCNL is seemingly a hazardous endeavor, with a high-risk of severe bleeding and acute or long-term renal function deterioration. For solitary kidney, whether congenital or acquired, the structure and histology of the kidney undergo compensatory changes, primarily manifested as increased renal volume, dilation of the afferent arterioles, elevated pressure, and increased glomerular filtration rate (GFR) (16). The increase in filtration and enhanced function of the kidney tubules facilitate the compensation of total GFR, such that the GFR of a single kidney is relatively the same as that of two kidneys. In the long term, this condition leads to glomerular damage, tubular injury, endothelial dysfunction, and ultimately results in renal dysfunction and chronic kidney disease (17). Although previous literature has presented some inconsistencies in conclusions regarding PCNL for stone management in solitary kidneys, the majority of studies support its safety and feasibility. Singh et al. (18) found that PCNL in solitary functioning kidney (SFK) patients is safe; however, patients with advanced renal insufficiency (decompensated stage) had a higher risk of severe complications such as dialysis. Torricelli et al. (19) concluded that PCNL in solitary kidney patients achieves satisfactory stone-free rates (SFRs) with low major complication rates, though multiple tract access may increase bleeding risks. Karkin et al. (20) reported that miniaturized PCNL (mPCNL) is safe and effective for pediatric renal stones. Additionally, Shi et al. (21) demonstrated that patients with solitary kidneys are more prone to acute kidney injury (AKI) compared to those with bilateral kidneys. The Doppler ultrasound mode offers advantages in detecting renal blood flow and visualizing significant vessels within puncture tracts. Lu et al. (22) demonstrated that Doppler ultrasound reduces the risk of vascular injury and hemorrhagic complications during renal puncture compared to conventional B-mode ultrasound, particularly in patients with solitary kidney stones. Furthermore, Tzeng et al. (9) conducted a prospective randomized controlled trial (RCT) revealing that Doppler-guided puncture was associated with fewer bleeding incidents. Similarly, Shi et al. (23) reported a significant reduction in severe hemorrhagic complications with the use of Doppler ultrasound technology.

To the best of our knowledge, this is the first comparative study evaluating the application of Doppler ultrasound in PCNL for solitary kidney stones. Our findings indicate that, compared to conventional ultrasound guidance, Doppler ultrasound showed no significant superiority in reducing intra-operative blood loss or lowering complication rates. Instead, it prolonged puncture time. Moreover, no significant differences were observed between the two groups in terms of operative duration, number of tracts, total renal function or SFR. In the conventional ultrasound group, 3 patients required postoperative blood transfusion, whereas 2 patients in the Doppler group received transfusions. Notably, three of these transfusions were administered to correct pre-existing renal anemia. Fortunately, none of the patients required selective renal artery embolization and all achieved full recovery. Postoperative 24-hour renal function tests revealed levels comparable to preoperative values, which might be attributed to the counteracting effects of obstruction relief against early renal functional elevation caused by surgical trauma.

The clarity of ultrasound images is influenced by numerous factors. For instance, the depth of the target organ can degrade image resolution as the scanning distance increases—a phenomenon particularly pronounced in obese patients and those with horseshoe kidneys or ectopic kidneys, which further compromises the accuracy of Doppler blood flow measurements (24). Additionally, respiratory motion and uneven pressure on the probe may distort or destabilize blood flow signals, potentially leading to a false positive or false-negative results. Moreover, smaller-diameter vessels, such as arcuate arteries and interlobular arteries, are challenging to detect precisely in Doppler imaging and are prone to injury during percutaneous renal puncture. In contrast, larger segmental renal vessels or tertiary branches, typically located within the renal columns, can generally be avoided by leveraging anatomical visualization via B-mode ultrasound imaging. Recently, contrast-enhanced ultrasound (CEUS) has been well-established and introduced in many diseases (25). It has also proven useful for pre-procedure planning, procedure guidance, needle-driven procedures, which illustrate the many benefits of CEUS-core needle biopsy (CNB) and percutaneous hepatic lesion ablation. Perhaps in the future, this technology could also be applied to percutaneous renal puncture procedures, enabling precise detection of more small arteries along the surgical pathway and thereby providing more accurate assistance in avoiding vascular injuries.

This study is subject to several limitations. First, the retrospective design inherently introduces potential selection bias due to uncontrolled confounding variables. Second, procedural standardization was influenced by operator-dependent nuances, as surgeries were exclusively performed by senior urologists (cumulative PCNL experience >3,000 cases) with mastery of sonographic renal mapping techniques. This expertise-driven procedural homogeneity limits the extrapolation of findings to broader clinical contexts, particularly in training environments where proficiency in ultrasound-guided anatomical navigation remains developmental. Third, the limited number of patients (76 cases) included in this study may impact the final research findings, low complication rate necessitates more patients to detect clinically meaningful differences. Fourth, follow-up protocols were confined to a short-term postoperative interval (30 days), neglecting longitudinal assessments of renal function evolution (>6-month) and secondary outcomes such as delayed complications.

## Conclusion

Our study shows ultrasound-guided PCNL is safe and effective in patients with solitary kidney stones, however, it offers no definitive advantage in hemorrhage prevention over conventional US guidance despite prolonged puncture times. While Doppler remains a valuable training tool for novices, its routine use may not be justified in experienced hands.

## Data Availability

The original contributions presented in the study are included in the article/Supplementary Material, further inquiries can be directed to the corresponding author.
